# Prevalence of *Candida albicans* and non-*albicans* isolates from vaginal secretions: comparative evaluation of colonization, vaginal candidiasis and recurrent vaginal candidiasis in diabetic and non-diabetic women

**DOI:** 10.1590/1516-3180.2014.1322640

**Published:** 2014-04-01

**Authors:** Luciene Setsuko Akimoto Gunther, Helen Priscila Rodrigues Martins, Fabrícia Gimenes, André Luelsdorf Pimenta de Abreu, Marcia Edilaine Lopes Consolaro, Terezinha Inez Estivalet Svidzinski

**Affiliations:** I MSc. Professor, Department of Clinical Analysis and Biomedicine, Universidade Estadual de Maringá (UEM), Maringá, Paraná, Brazil; II MSc. Nurse, Municipal Health Department of Curitiba, Curitiba, Paraná, Brazil; III PhD. Postdoctoral Student, Department of Clinical Analysis and Biomedicine, Universidade Estadual de Maringá (UEM), Maringá, Paraná, Brazil; IV MSc. Postgraduate Doctoral Student, Department of Clinical Analysis and Biomedicine, Universidade Estadual de Maringá (UEM), Maringá, Paraná, Brazil; V PhD. Professor, Department of Clinical Analysis and Biomedicine, Universidade Estadual de Maringá (UEM), Maringá, Paraná, Brazil

**Keywords:** Candida, Candidiasis, vulvovaginal, Diabetes mellitus, Fluconazole, Therapeutics, Candida, Candidíase vulvovaginal, Diabetes mellitus, Fluconazol, Terapêutica

## Abstract

**CONTEXT AND OBJECTIVE:**

Vulvovaginal candidiasis (VVC) is caused by abnormal growth of yeast-like fungi on the female genital tract mucosa. Patients with diabetes *mellitus* (DM) are more susceptible to fungal infections, including those caused by species of *Candida*. The present study investigated the frequency of total isolation of vaginal *Candida* spp., and its different clinical profiles - colonization, VVC and recurrent VVC (RVVC) - in women with DM type 2, compared with non-diabetic women. The cure rate using fluconazole treatment was also evaluated.

**DESIGN AND SETTING:**

Cross-sectional study conducted in the public healthcare system of Maringá, Paraná, Brazil.

**METHODS:**

The study involved 717 women aged 17-74 years, of whom 48 (6.7%) had DM type 2 (mean age: 53.7 years), regardless of signs and symptoms of VVC. The yeasts were isolated and identified using classical phenotypic methods.

**RESULTS:**

In the non-diabetic group (controls), total vaginal yeast isolation occurred in 79 (11.8%) women, and in the diabetic group in 9 (18.8%) (P = 0.000). The diabetic group showed more symptomatic (VVC + RVVC = 66.66%) than colonized (33.33%) women, and showed significantly more colonization, VVC and RVVC than seen among the controls. The mean cure rate using fluconazole was 75.0% in the diabetic group and 86.7% in the control group (P *= *0.51).

**CONCLUSION:**

We found that DM type 2 in Brazilian women was associated with yeast colonization, VVC and RVVC, and similar isolation rates for *C. albicans* and non-*albicans* species. Good cure rates were obtained using fluconazole in both groups.

## RESUMO

**CONTEXTO E OBJETIVO:**

Candidíase vulvovaginal (CVV) é causada pelo crescimento anormal de fungos do tipo leveduras na mucosa do trato genital feminino. Pacientes com diabetes *mellitus* (DM) são mais susceptíveis a infecções fúngicas, incluindo por espécies de *Candida*. O presente estudo investigou a frequência de isolamento total de *Candida *spp. vaginal, e diferentes quadros clínicos (CVV e CVV recorrente- CVVR) em mulheres com DM tipo 2 comparadas às não diabéticas. A razão de cura do tratamento com fluconazol também foi avaliada.

**TIPO DE ESTUDO E LOCAL:**

Estudo transversal realizado no sistema público de saúde de Maringá, Paraná, Brazil.

**MÉTODO:**

O estudo envolveu 717 mulheres de 17-74 anos de idade e, destas, 48 (6,7%) tinham DM 2 (média de 53,7 anos), independentemente de sinais e sintomas de CVV. As leveduras foram isoladas e identificadas por métodos fenotípicos clássicos.

**RESULTADOS:**

No grupo de não diabéticas (controle), leveduras vaginais totais foram isoladas em 79 (11,8%) mulheres, e no grupo de diabéticas, em 9 (18,8%) (P = 0,000). O grupo de diabéticas mostrou mais mulheres sintomáticas (CVV + CVVR = 66,66%) do que colonizadas (33.33%), e significativamente mais colonização, CVV e CVVR, que as controle. A razão média de cura com fluconazol foi de 75.0% no grupo diabéticas e 86.7% no controle (P *=* 0.51).

**CONCLUSÃO:**

Nós encontramos que DM 2 em mulheres brasileiras associou-se com colonização vaginal por leveduras, CVV e CVVR, razão similar de isolamento de *C. albicans *e espécies não *albicans*. Boa taxa de cura foi obtida com fluconazol em ambos os grupos.

## INTRODUCTION

Vulvovaginal candidiasis (VVC) is classified by the World Health Organization (WHO) as a pathological condition that is frequently sexually transmitted (STD).^1^ Because it affects millions of women annually, thereby causing great discomfort, interfering with sexual and affective relations and impairing work performance, VVC has been considered to be an important worldwide public health problem.^2^

VVC is caused by abnormal growth of yeast-like fungi on the mucosa of the female genital tract. It is clinically characterized by occurrences of intense vulvar itching, leucorrhea, dyspareunia, dysuria, edema and vulvovaginal erythema.^2^ ^,^ ^3^ Vaginal yeasts become pathogenic when the colonization site on the host is favorable to their development. Several factors may increase this risk, such as previous colonization by the yeast, immunosuppressive diseases, diabetes *mellitus* (DM) and other factors.^4^ ^,^ ^5^

Patients with DM are more susceptible to bacterial and fungal infections, including those caused by species of *Candida*.^6^ Some investigators have suggested that VVC occurs more frequently in diabetic women, and others that a correlation exists between hyperglycemia and VVC.^2^ ^,^ ^7^ However, few studies have addressed the problem of VVC among Brazilian diabetic women.

## OBJECTIVE

The objective of this study was to determine the frequency of total isolation of vaginal Candida spp., and the clinical profiles, VVC and recurrent VVC (RVVC), in women with DM type 2, compared with non-diabetic women. The cure rate from fluconazole treatment was also evaluated.

## METHODS

This experimental study involved women aged between 17 and 74 years who participated in the Cervical Cancer Triage Program, regardless of signs and symptoms of VVC, between January 1 and December 31, 2010, in the public healthcare system of Maringá, Paraná, Brazil. Six healthcare centers participated in the study. This study was approved by the Ethics Committee for Research on Humans (COPEP) at Universidade Estadual de Maringá (UEM) (185/2007). The exclusion criteria were pregnancy or a history of immunosuppressive disease, including AIDS. The women answered a standardized questionnaire that sought information regarding VVC symptoms. Subjects were identified as affected by DM type 2 according to the American Diabetes Association (ADA) definition, if their fasting serum glucose was 7 mmol/l (126 mg/dl) or more, as reported in the patients' medical records.

A vaginal sample was collected using a sterile swab, inoculated in sterile saline and sent to the Medical Mycology Laboratory at UEM, where it was immediately seeded onto plates containing Sabouraud dextrose agar (SDA) (Difco, United States) with 100 mg/ml of chloramphenicol, and incubated at 25 °C for five days. A pool of the colonies grown on each plate was subcultured in CHROMagar Candida medium (Probac, France). Beginning with the pure culture, the yeasts were identified using classical phenotypic methods.^8^

The clinical profiles of the women with positive culture for yeasts were classified into three types: colonized, but without symptoms of VVC; with VVC, presenting an acute episode with at least two of the following symptoms: discharge, itching, dysuria and dyspareunia; and with recurrent VVC (RVVC), presenting two or more of these symptoms and at least four episodes in twelve months.^9^ Women who had RVVC were counted only once.

All the women in the diabetic group and 28 in the non-diabetic group with cultures positive for yeasts were treated, independent of clinical profile, using oral fluconazole (Neoquímica, Brazil) at a single dose of 150 mg weekly for two weeks. They were instructed to return 20 days after the end of treatment, so that material for a new yeast culture could be collected.

The data were analyzed by means of the chi-square test, using the STATA for Statistics and Data Analysis 9.1 software. All variables were expressed as absolute and relative frequencies. P values < 0.05 were considered significant.

## RESULTS

**Figure 1:**
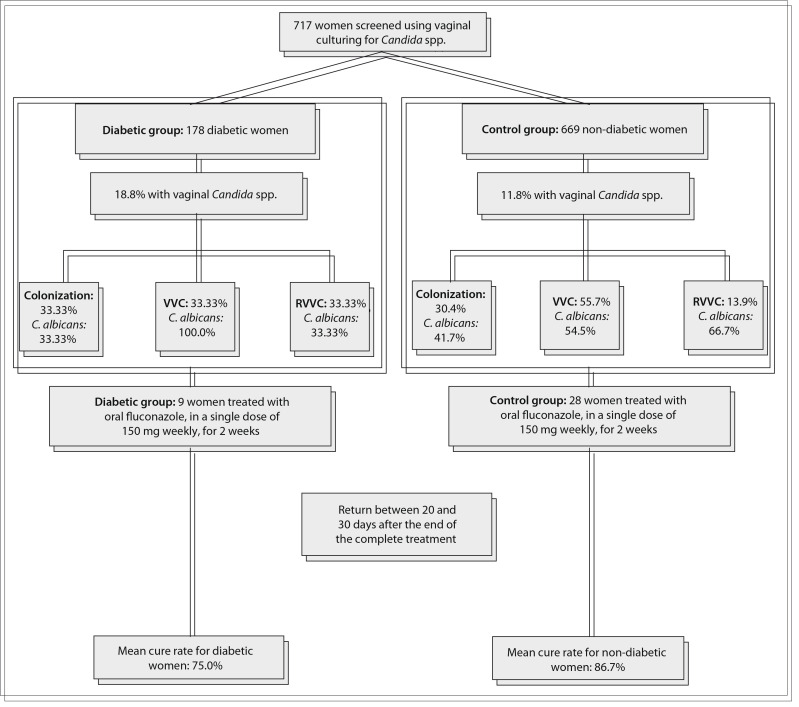
An overview of the study and its results

Figure 1 shows an overview of the study. A total of 717 women were included, of whom 48 (6.7%) had DM type 2 (mean age: 53.7 years). In the control group (mean age: 43.3 years), total vaginal yeast isolation occurred in 79 (11.8%) women, distributed as C. albicans (n = 43; 54.4%) and non-albicans species (n = 36; 45.6%) (odds ratio [OR] = 7; 2.2-11.5; P = 0.02). In no case was more than one yeast species isolated.

In the diabetic group, total yeast isolation occurred in 9 women (18.8%), which was a higher proportion than among the controls (OR = 7.77; 3.88-15.56; P = 0.000), distributed as *C. albicans *(n = 5; 55.6%) and non*-albicans* species (n = 4; 44.4%) (P = 0.2) (Table 1).

**Table 1:** Frequency of total isolation of vaginal Candida spp. and the clinical conditions of colonization, vulvovaginal candidiasis (VVC) and recurrent VVC (RVVC) among diabetic and non-diabetic women in the public healthcare system of Maringá, Paraná, Brazil

*Candida* species	Diabetic group (n = 48)							Non-diabetic group (n = 669)								
	Total isolation^*^		Colonization^*^		VVC^*^		RVVC^*^		Total isolation		Colonization		VVC		RVVC	
	n	%	n	%	n	%	n	%	n	%	n	%	n	%	n	%
*C. albicans *	5	55.6	1	33.3	3	100.0	1	33.3	43^†^	54.4	10	41.7	24	54.5	9	66.7
Non-*albicans *	4	44.4	2	66.7	-	-	2	66.7	36	45.6	14	58.3	20	45.5	2	33.3
Total	9	18.8	3	33.3	3^‡^	33.3	3^‡^	33.3	79	11.8	24^§^	30.4	44^§^	55.7	11^§^	13.9

n = number

VVC = vulvovaginal candidiasis

RVVC = recurrent vulvovaginal candidiasis

* Total Candida spp. isolation (clinical conditions: colonization, VVC and RVVC) was significantly greater in the diabetic group (P = 0.000)

†C . albicans was the most frequent species isolated in the non-diabetic group (P = 0.02) considering only total Candida spp. isolation, but not for different clinical conditions (colonization, VVC and RVVC) (P > 0.05)

‡ Diabetic group showed more symptomatic women (VVC and RVVC) than colonized women (odds ratio, OR = 0.5

§ Control group was equally colonized or had VVC or RVVC (P = 0.411), without significant difference between VVC and RVVC (P = 0.201). Diabetic group showed more colonization (OR = 5; 2.08-23.46; P = 0.005)

VVC + RVVC (OR = 5; 2.50-10.44; P = 0.004)

VVC (OR = 13; 4.23-49.13; P = 0.000)

RVVC (OR = 2.6; 0.70-10.05; P = 0.000) than seen in the controls.

Among the non*-albicans* species, *C.* *glabrata *was the most frequent isolate in both the controls (n = 23/79; 29.1%) and the diabetic group (n = 3/9; 33.3%).

With regard to clinical profiles, in the control group 24/79 women (30.4%) were colonized or had VVC or RVVC (55/79; 69.9%) (P = 0.411), with no significant difference between VVC (44/79; 55.7%) and RVVC (11/79, 13.9%) (P = 0.201). In the diabetic group, more women were symptomatic (VVC + RVVC = 3/9, 66.66%) than colonized (3/9; 33.33%) (OR = 0.5; 0.125-1.999; P = 0.005) (Table 1). 

The diabetic women showed a significantly higher proportion of colonization (OR = 5; 2.08-23.46; P = 0.005), VVC + RVVC (OR = 5; 2.50-10.44; P = 0.004), VVC (OR = 13; 4.23-49.13; P = 0.000) and RVVC (OR = 2.6; 0.70-10.05; P = 0.000) than seen in the controls.

A total of 8 women in the diabetic group and 15 in the non-diabetic group correctly concluded the treatment with fluconazole, with mean cure rates of 75.0% and 86.7% respectively (P = 0.51). In both the diabetic and the control group, *C. glabrata* and *C. tropicalis *showed resistance to fluconazole. 

## DISCUSSION

We found that DM type 2 in Brazilian women was associated with Candida spp. colonization, VVC and RVVC; and that the cure rate with fluconazole was satisfactory. Other investigators have described associations between DM and VVC, colonization and RVVC, in different countries.^6^ ^,^ ^10^ This is very worrisome because VVC afflicts millions of both non-diabetic and diabetic women, causing great discomfort and interfering with sexual and affective relations.^2^ ^,^ ^3^ Uncontrolled DM causes metabolic alterations, such as increased levels of glycogen, which can significantly increase colonization and infection by Candida.^11^ The increased glycogen level lowers the vaginal pH, thereby facilitating development of VVC.^12^

The women with DM studied here showed similar rates of *C. albicans* and non-*albicans* species, thus differing from the control group. The rates were similar to those reported by Lattif et al.^10^ and Faraji et al.^13^ In some populations, there has been an increase in the isolation of vaginal non-*albicans* species, but most investigators agree that this does not seem to be a general trend.^10^ ^,^ ^13^

The relatively high cure rate with fluconazole in both diabetic and non-diabetic women shows that this is a good therapeutic option even for Brazilian women with diabetes, who require attention to treatment of non-*albicans* species, particularly *C. glabrata*, which are intrinsically less susceptible to azole antifungals.^3^ ^,^ ^14^ Similarly to our results, Brumar et al.^7^ also reported a high cure rate with fluconazole (85.71%) in diabetic women with VVC. 

We acknowledge that the number of diabetic women in our study was small, and that this group may not fully represent populations of diabetic women. Nevertheless, the number of diabetics (n = 48) was relatively high in this population of 717 women, and we believe that this study contributes important information for management of diabetic women with vaginal *Candida* spp. However, the interaction between DM type 2 and vaginal *Candida *species merits further evaluation in relation to glycemic control, in a larger sample of diabetic women in Brazil. 

## CONCLUSION

We found that diabetes in Brazilian women was associated with yeast colonization, VVC and RVVC, with similar isolation rates for C. albicans and non-albicans species; and that the cure rate with fluconazole was relatively high. Although regarded as a trivial infection by some, the increasing incidence of VVC associated with diabetes raises additional issues regarding prevention and patient management.
